# Effect of Preoperative Zoledronic Acid Administration on Pain Intensity after Percutaneous Vertebroplasty for Osteoporotic Vertebral Compression Fractures

**DOI:** 10.1155/2020/8039671

**Published:** 2020-08-03

**Authors:** Weiran Hu, Hongqiang Wang, Xinge Shi, Yuepeng Song, Guangquan Zhang, Shuai Xing, Kai Zhang, Yanzheng Gao

**Affiliations:** ^1^Department of Spinal Cord Surgery, Henan Provincial People's Hospital, Zhengzhou, Henan 45003, China; ^2^People's Hospital of Zhengzhou University, Zhengzhou, Henan 45003, China

## Abstract

**Introduction:**

This study aimed to compare and analyze the effect of preoperative zoledronic acid (ZOL) administration on pain intensity after percutaneous vertebroplasty (PVP) for osteoporotic vertebral compression fracture (OVCF).

**Methods:**

The study included 242 patients with OVCFs who underwent PVP in our hospital between January 2015 and June 2018. The patients were randomly assigned to either a ZOL group (*n* = 121) or a control group (*n* = 121). The patients in the ZOL group were treated preoperatively with intravenous infusion of 5 mg ZOL. Those in the control group were treated without ZOL. All the patients were followed up for 1 year.

**Results:**

No statistically significant differences in age, sex, weight, and body mass index (BMI) were found between the two groups. During the follow-up period, the visual analog scale score and Oswestry dysfunction index score in the ZOL group were lower than those in the control group. The bone mineral density at 6 or 12 months after treatment was significantly higher and the levels of the bone metabolism markers were significantly lower in the ZOL group than in the control group (*P* < 0.05 for both). Two patients in the treatment group had new vertebral fractures, whereas 13 patients in the control group had new vertebral fractures, which translate to recompression vertebral fracture incidence rates of 1.7% and 10.7%, respectively. The incidence rate of mild adverse reactions was significantly higher in the ZOL group than in the control group, but all the cases were endurable.

**Conclusion:**

Intravenous infusion of ZOL before PVP can effectively reduce postoperative pain intensity, reduce bone loss, increase bone density, reduce the risk of refracture, and improve patient quality of life.

## 1. Introduction

Osteoporotic vertebral compression fracture (OVCF) is one of the most common fractures in patients with osteoporosis. However, most OVCFs are stable and asymptomatic vertebral fractures that do not require open surgical therapy. Compared with the traditional conservative treatment, percutaneous vertebroplasty (PVP) is characterized by fewer complications, positive efficacy, and less trauma. Immediate analgesia can be achieved by fixing the broken end of the fracture, and the operation can enhance the strength and stiffness of the vertebral body, restore the height of the vertebral body, and correct the deformity in kyphosis.

However, PVP still has some problems such as postoperative residual pain in the lower back and the development of new vertebral fractures, which may cause other problems in patients. A study by Zhong et al. suggested that 12.9% of patients had new fractures within 1 year after PVP [1]. Zoledronic acid (ZOL) administration can effectively increase lumbar bone density and reduce the risk of vertebral fracture in patients with osteoporosis, which has been reported in the literature [2]. Thus, it may have great significance in consolidating the surgical treatment effect of PVP and preventing new vertebral fractures.

To test this hypothesis, our department adopted the use of ZOL in combination with PVP surgery to improve the treatment of OVCF. To verify the effectiveness of this method, 242 patients who underwent PVP between January 2015 and June 2018 were randomly divided into two groups. In the treatment group, ZOL and calcium supplements were administered 2 days before the PVP surgery. In the control group, PVP was performed after calcium supplementation only. All the patients were followed up for 1 year, and the changes in visual analog scale (VAS) score, Oswestry dysfunction index (ODI) score, lumbar bone density, and bone metabolism index scores were observed and calculated.

## 2. Materials and Methods

### 2.1. General Information

The patient inclusion criteria for the study were as follows: patients with a clear medical history and clinical diagnosis of OVCF, patients who underwent imaging examination to assist diagnosis, and patients whose radiography and computed tomography findings suggested the presence of osteoporosis and changes in vertebral volume compressibility. Magnetic resonance imaging revealed a high signal intensity on the T2-weighted image of the diseased vertebral body, which confirmed the diagnosis of fresh vertebral compression fracture. Bone mineral content was determined in accordance with the *American Association of Clinical Endocrinologists and American College of Endocrinology Clinical Practice Guidelines for the Diagnosis and Treatment of Postmenopausal Osteoporosis 2016* [3]. According to the reference standard, osteoporosis was diagnosed when the *T* value of the femoral neck was <−2.5, and osteopenia, when the *T* value was between −1.0 and −2.0.

The exclusion criteria were as follows: patients with vertebral blowout fractures, intraspinal occupation, or neurological symptoms; patients with severe neurological and psychiatric disorders, who were lost to follow-up, and who were incapable of undergoing follow-up tests; patients with chronic liver and kidney function damages; patients with severe digestive diseases; patients with thyroid gland and parathyroid gland diseases; and patients with malignant tumor metastasis and long-term use of glucocorticoid drugs.

### 2.2. Grouping and Methods

After hospital admission, 242 patients, including 125 men and 117 women, were randomly divided into two groups. The mean age was 69.5 ± 6.8 years. The affected vertebrae were located at the *T*5–*L*5 levels, with a total of 367 vertebral bodies. According to the location of the fractured vertebrae, 89 fractures involved the thoracic vertebrae and 57 involved the lumbar vertebrae in the ZOL group, whereas 101 fractures were in the thoracic vertebrae and 51 were in the lumbar vertebrae in the control group. No statistically significant differences in baseline characteristics such as sex, age, height, and physical signs were found between the two groups.

Both groups received oral calcium carbonate/vitamin D3 tablets (600 mg/d; Wyeth Pharmaceuticals) since hospital admission until after surgery continuously. The ZOL group was treated with ZOL injection (5 mg/100 ml; Aclasta, Novartis Pharma Schweiz AG) 2 days before surgery, and no postoperative analgesia was used in both groups. The follow-up time points were 3 days, 1 month, 6 months, and 1 year after the operation (Table 1).

### 2.3. Observation Indicator

The observation indicators were as follows: (1) visual analog scale (VAS) score, where the patients rated their pain level on the relevant scale (0–10), with 0 indicating no pain and 10 indicating the most severe pain; (2) bone mineral density, where a dual-energy X-ray bone mineral density detector was used to detect the bone mineral density and the orthotopic bone mineral density and salt content of the femoral neck were measured; (3) Oswestry disability index (ODI) score, where the ODI, a specific scoring system for low back pain, has been widely used in the field of spinal surgery to investigate the degree of dysfunction according to 10 categories; and (4) bone markers, where *β*-CTX (*β*-isomerized C-terminal telopeptide of type I collagen) and P1NP (N-terminal propeptide of type I collagen) as bone markers were detected using the Cobas6000 E601 automatic immunoluminescence analyzer (Roche).

### 2.4. Statistical Analyses

The SPSS 18.0 statistical software was used for the statistical analysis. Quantitative data were expressed as *x* ± *s*. A *t*-test was used for comparison between groups, and a paired *t*-test was used before and after treatment. Results with *P* values of <0.05 were considered significantly different.

## 3. Results

### 3.1. Pain Improvement

**Figure 1:**
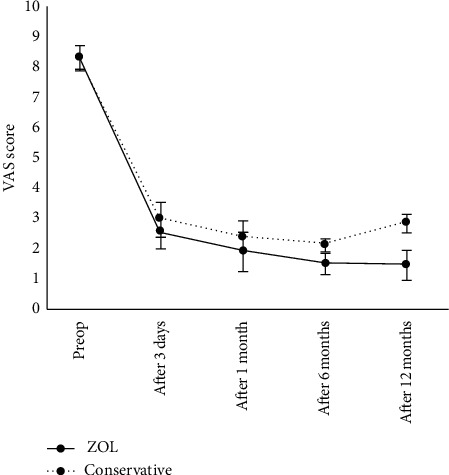
Visual analog scale (VAS) scores before and after percutaneous vertebroplasty (PVP) and/or zoledronic acid (ZOL) infusion. Data are presented as mean ± SD.

No significant difference in pretreatment VAS score was found between the ZOL and control groups (*P* < 0.05). However, the VAS score significantly differed between the two groups (*P* < 0.01) during the follow-up period. In the ZOL group, the postoperative VAS score significantly decreased gradually from that before operation (*P* < 0.01). The VAS score of the control group after the 6-month follow-up was slightly higher than that of the ZOL group but lower than that before operation (Figure 1).

### 3.2. Changes in ODI Score

**Figure 2:**
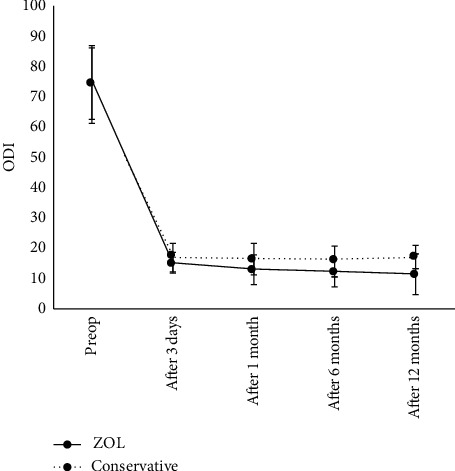
Oswestry dysfunction index (ODI) before and after percutaneous vertebroplasty (PVP) and/or zoledronic acid (ZOL) infusion. Data are presented as mean ± SD.

No statistically significant differences in ODI scores before treatment and 3 days after surgery were found between the two groups (*P* < 0.05). At 1 month and 6 months after operation, all the activity functions of the patients in the two groups were improved as compared with those before the operation, and the differences were statistically significant (*P* < 0.01). The ODI score of the ZOL group was still significantly decreased 6 months after surgery as compared with 1 month after surgery (*P* < 0.01). In the control group, the ODI score showed no statistically significant difference between 1 month and 6 months after surgery (*P* < 0.05; Figure 2).

### 3.3. Changes in Bone Density

The comparison between the two groups showed no significant difference in left femoral neck bone mineral density before treatment (*P* < 0.05). All the patients were followed up at 6 and 12 months after surgery. The results showed that bone density increased in both groups but was statistically significantly higher in the ZOL group than in the control group at 6 or 12 months after treatment. The intragroup comparison revealed that, in the treatment group, the femoral neck mineral density at the 12-month follow-up was significantly higher than those at 6 months and before operation (*P* < 0.01; Table 2).

### 3.4. Changes in Bone Metabolic Factors

No significant difference in serum P1NP and *β*-CTX levels was found between the two groups before treatment (*P* < 0.05). Continuous monitoring after treatment revealed that the serum *β*-CTX and P1NP levels decreased and were significantly lower in the ZOL group than in the control group during follow-up (*P* < 0.01). In the ZOL group, the PINP and *β*-CTX levels decreased during the first 6 months after operation and increased 6 months after operation but remained at lower levels than those in the control group (Table 3).

### 3.5. New Vertebral Fracture

According to the statistical data at 12 months of follow-up, 2 patients (1 man and 1 woman) in the treatment group had new vertebral fractures in a total of 3 vertebral bodies, whereas 13 patients (4 men and 9 women) in the control group had new vertebral fractures in a total of 17 vertebral bodies.

### 3.6. Complications

Twenty-three patients complained of discomfort after ZOL administration, including 21 cases (17.4%) of fever, 17 cases (14.0%) of influenza-like symptoms, and 9 cases (7.4%) of muscle and soft tissue pain. Eight cases of bone cement leakage occurred in the experimental group; and 10 cases, in the control group, all of which showed no invasion of the spinal canal. We found no statistically significant difference in bone cement leakage rate between the two groups (*P* < 0.05; Table 4).

## 4. Discussion

Osteoporotic fracture (OF) has become an international public health problem and is the most severe complication of osteoporosis, characterized by high morbidity, disability, mortality, and medical costs. Among all of cases, OVCF accounts for the largest proportion. Without positive intervention, vertebral lesions may lead to imbalance in the sagittal plane of the spine, causing a chain fracture reaction in other vertebral bodies, accelerate the hump, and ultimately lead to severe kyphosis, which seriously affect patient quality of life. Previous studies reported that >39% of women aged >65 years had OVCFs [4–7].

PVP is mainly used in the treatment of patients with osteoporotic VCF. This technology has the advantages of immediate analgesic effect, limited increase in vertebral height, improvement of spinal deformity, and increased vertebral stability. It has become the currently recommended treatment method for OVCF. Through a cohort study, Yang et al. found no significant difference in VAS and ODI scores between the PVP and conservative treatment groups after 6 months, but PVP could rapidly reduce pain and restore daily life activities at an early date [4]. In a meta-analysis of 13 randomized controlled trials with 1624 patients, Lou et al. concluded that PVP is safe and effective for rapid pain relief in patients with acute OVCF [5]. A study by Wang et al. suggested that PVP can significantly improve postoperative pain in patients with OVCF as compared with facet arthroplasty [6]. In a meta-analysis, Zhang et al. also concluded that compared with conservative treatment, PVP significantly reduced pain and improved the quality of life of patients while reducing the risk of re-fracture [7].

In this study, VAS score was reduced, pain was relieved, and quality of life was significantly improved after PVP surgery in both groups. However, with the extended follow-up time, the increasing trend of the VAS score was more obvious in the control group than in the ZOL group. We believe that this may be related to the residual pain caused by osteoporosis and the new vertebral fracture after surgery. Tan et al. conducted a prospective study on chronic pain caused by OVCF. After 1 year of follow-up after PVP treatment, the patients' back pain symptoms were significantly relieved. We believe that PVP is effective for relieving chronic pain caused by OVCF [8]. Zhang et al. reported that the VAS score of the patients with OVCF decreased from 7.6 ± 0.78 to 2.45 ± 0.51 after PVP treatment, indicating satisfactory surgical results [9].

The main mechanisms thereby PVP relieves low back pain are as follows. (1) Bone cement polymerization and solidification release a large amount of heat that cauterizes nerve endings. (2) Bone cement solidifies the fracture pieces together, increases the stability of the vertebral body, and reduces the stimulation of fracture tablets. (3) Bone cement can embolize local blood vessels, resulting in peripheral nerve ischemia and necrosis and thereby achieving an analgesic effect. Postoperative low back pain was significantly relieved in all the patients, and the postoperative VAS score was significantly reduced in both groups. A study by Ma et al. showed that PVP could alleviate pain in patients with OVCF in the early stage, partially restore the vertebral height, and significantly improve the VAS score in the first 1–3 months after surgery [10]. Ge et al. proposed that after 36 months of follow-up, radiography and VAS scores were used to evaluate patient prognosis. The authors believed that PVP treatment of OVCF was safe and effective and could quickly relieve low back pain, restore the height of fractured thoracic vertebrae, correct kyphosis, and improve the quality of life of patients [11]. Wang et al. retrospectively evaluated 35 patients with severe OVCF and found that pain was significantly relieved after PVP treatment. The authors believe that PVP for OVCF is a safe and effective treatment that can significantly restore vertebral height, reduce the kyphosis angle, significantly relieve pain, and improve limb function [12]. Clarençon retrospectively analyzed the safety and clinical efficacy of PVP in 173 patients aged >80 years who had OVCFs. They found that 79.3% of the elderly patients attained pain relief after PVP and thus concluded that PVP is a safe treatment option for elderly patients [13].

However, most patients still have mild residual pain after surgery. Some scholars believe that PVP only relieves acute pain caused by the fracture but fails to relieve the pain caused by osteoporosis. At the same time, some scholars believe that the postoperative strength of the vertebral body with compression fracture increases, changing the mechanical structure and transmission mechanism of the normal vertebral bodies, aggravating the load of the adjacent vertebral bodies, and thus increasing the risk of fracture of the adjacent vertebral bodies or causing occult trabecular bone fractures in the adjacent vertebral bodies, which will result in postoperative residual pain [14]. Yang et al. performed a statistical analysis for 1316 patients treated with PVP, among which 60 complained of postoperative discomfort, with a prevalence of 4.6%. The analysis result suggested that low bone density, lumbar fascia injury, multisegment PVP, insufficient injection volume of bone cement, unsatisfactory distribution of bone cement, and depression were important factors of postoperative residual pain in patients with OVCF [15]. A prospective cohort study led by Yan et al. included 133 elderly patients with OVCF. VAS score and ODI were used to evaluate postoperative efficacy, and fascia injury was identified as an important cause of postoperative residual lumbago and back pain [16].

The special double nitrogen side chain structure of ZOL has a high affinity for bone tissue, which can selectively act on osteoclasts, inhibit the activity of osteoclasts, inhibit bone absorption, slow down bone loss, and increase bone mass [17]. ZOL has the advantages of long-acting, obvious, and fast-acting effect, and significantly improving bone density. A multicenter, randomized, double-blind, controlled trial that administered ZOL and placebo at 6, 12, 18, and 24 months ultimately concluded that ZOL administration reduced bone mass loss and pain [18]. Cai et al. conducted a trial in patients aged >40 years who had low back pain and vertebral modic changes for 6 months. Compared with that of the placebo group, the VAS score of the ZOL group decreased significantly [19]. Liu et al. analyzed the clinical data of 482 elderly patients with osteoporotic fractures. The VAS score, bone mineral density, and incidence of recurrent fracture were better in the ZOL group after 24 months of ZOL treatment than in the control group. The authors believe that ZOL administration can reduce postoperative bone mass loss and recurrent fracture in patients with osteoporotic fractures [20]. In this study, the bone mineral density in the ZOL group was significantly improved at 12 months after surgery. However, the VAS scores in the control group increased. Therefore, in this study, we concluded that PVP combined with ZOL administration was superior to PVP alone in terms of pain relief, and the difference in pain relief between the two groups increased gradually during follow-up.

No significant difference in ODI measured at 3 days after surgery was found between the two groups, indicating that the immediate postoperative pain relief was the same between the two groups. However, with the increase in follow-up time, the ODI score of the ZOL group continued to decline, indicating that the patients' waist function continued to recover, whereas that of the control group did not continue to improve, and the difference between the two groups gradually emerged. In the experimental group, after intravenous ZOL administration, osteoporosis continued to improve and effectively relieved the postoperative residual pain caused by osteoporosis. In the control group, only the broken ends of the fractures were fixed through surgery, but no other treatment was performed for osteoporosis. Hu et al. examined 72 patients aged >60 years who had OVCFs. After measuring their spinopelvic parameters, they concluded that the spinal sagittal imbalance and ODI score were higher in the patients with OVCF than in the control group, seriously affecting quality of life [21]. Wang et al. followed up the clinical efficacy of postoperative PVP. During the follow-up of 43 patients, the mean ODI score of the patients decreased from 40 to 8 at 6 months after surgery [22].

Among the bone metabolism markers, *β*-CTX, a carboxy-terminal degradation product of collagen type I, is released into the blood circulation during osteoclast absorption of the bone matrix, which is a good indicator of bone resorption activity. P1NP is a bone-forming marker that reflects changes in newly synthesized collagen type I. The decrease in *β*-CTX level in our study suggested that ZOL had a stable long-term inhibitory effect on bone resorption and could effectively inhibit the activity of osteoclasts [23]. The P1NP level was lower after ZOL treatment, which suggests that ZOL could inhibit osteoclasts and reduce the levels of bone resorption markers [24]. In this study, the serum concentrations of PINP and *β*-CTX in the treatment group were lower than those in the control group within 12 months after treatment, which suggests that compared with those in the control group, bone formation and bone absorption were reduced in the ZOL group. Although the concentrations of PINP and *β*-CTX in the ZOL group increased after 3 months, they still remained low and, at the end of follow-up, were still less than half of the preoperative concentrations. This indicates that, after 1 year of administration, ZOL treatment still had a good inhibitory effect on bone conversion. From the pathophysiological perspective, this also explains that the relief of low back pain in the ZOL group was better than that in the control group. Our experimental results were similar to those reported by Zhang et al. [25]. The authors retrospectively evaluated 101 patients with OVCF. After the use of ZOL, the concentrations of PINP and tab-CTX decreased from 39.98 ± 1.79 g/L and 0.55 ± 0.14 g/L to 15.40 ± 1.40 g/L, and 0.34 ± 0.05 g/L, respectively, after 6 months of follow-up.

**Figure 3:**
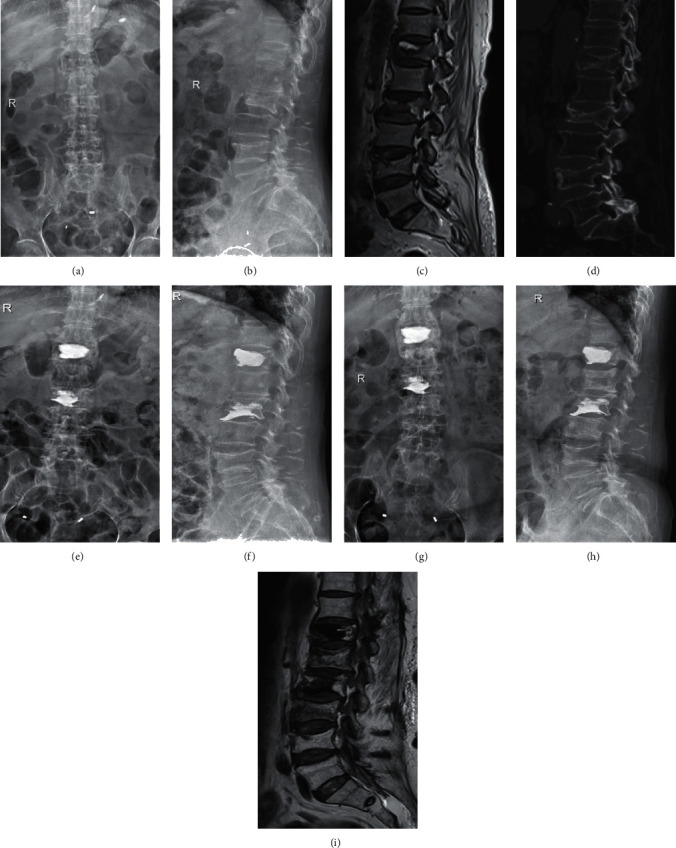
A 70-year-old woman. (a, b) Radiographs obtained in positive and lateral positions, showing a T12–L2 osteoporotic vertebral compression fracture. (c) Lumbar magnetic resonance image showing T12 and L2 vertebral body morphology changes. (d) Lumbar computed tomographic image showing T12 and L2 osteoporotic vertebral compression fractures. (e, f) Radiographs obtained in the positive and lateral positions after the first PVP operation for the T12 and L2 osteoporotic vertebral compression fractures. (g–i) At 8 months after the first treatment, the radiograph obtained in the positive and lateral positions and lumbar magnetic resonance image show new osteoporotic vertebral compression fractures at L1 and L3.

New vertebral fracture after PVP is also an important factor of pain, and a study reported that the incidence of new vertebral refracture after PVP surgery reached 10% [26]. Takahara et al. reported that the incidence of new vertebral fracture after PVP even reached 22.9% [27]. Many factors cause refracture after PVP, among which osteoporosis is the most important factor. The prevention of osteoporotic fractures should include guidance on appropriate exercise, proper diet, adjustment of lifestyle, and rational medication. In this study, 2 new vertebral fractures (3 vertebral bodies in total) occurred in the ZOL group (Figure 3), while 13 new vertebral fractures (17 vertebral bodies in total) occurred in the control group. This also partly explains the reason why the postoperative VAS score of the ZOL group was lower than that of the control group. The incidence of new vertebral fracture in the two groups was statistically significant (*P* < 0.05). The results showed that ZOL administration before surgery can effectively reduce the incidence of new vertebral fractures. Through a retrospective analysis, Yang et al. divided the time of surgery into within and after the 30 days after injury, analyzed the postoperative situation of new vertebral fractures, and found that the probability of new fractures in patients undergoing surgery within 30 days after injury was significantly lower than that in the control group [28]. Zhong et al. established a risk prediction model to simulate the risk factors of new vertebral fracture after PVP surgery and concluded that the independent risk factors of new vertebral fracture were intervertebral cement leakage and previous vertebral compression fracture [1]. Lee et al. conducted a retrospective cohort study that followed up 198 patients after PVP surgery, analyzed the risk factors of new vertebral fractures, and concluded that the osteoporosis treatment and improvements in BMD and BMI were the most important factors for reducing the risk of new vertebral fracture [29]. This is highly consistent with the conclusions of our study, and the incidence of vertebral fracture after PVP can be effectively reduced by improving bone density. In their recent study, Li et al. reached a similar conclusion. Patients received PVP with combined ZOL and rosuvastatin therapy. Between-group comparisons of bone density, type I procollagen peptide (CTX) and bone-specific alkaline phosphatase (BAP) levels, VAS score, ODI score, and adjacent centrum refracture were performed before and after treatment. Bone density was higher, and BAP and CTX levels, ODI score, and VAS score were lower in the observation group than in the control group. The refracture rate in the observation group was lower than that in the control group [30].

Complications of ZOL treatment, such as flu-like symptoms, fever, and fatigue, have been widely reported [31–33], which generally lasts around 3–7 days. At the same time, the side effects of ZOL administration are generally tolerable and can be quickly relieved by self-regulation. A multicenter study from China observed and analyzed the acute side effects of ZOL infusion in patients with osteoporosis. The incidence of fever within 7 days after ZOL infusion was 28.65% (740/2583), of which 98.34% (727/740) occurred within 5 days after infusion. Among the other side effects were pain in 312 patients (26.28%) or pain aggravation in 144 patients (10.18%), most of which occurred within 3 days of ZOL administration. These symptoms are usually mild to moderate, with a short duration, which makes ZOL treatment generally safe [33]. The same conclusion was reached in this study. Although the patients in the experimental group had side effects, the follow-up observation did not show serious damage in the patients, and the complications were eventually cured in all the patients. Therefore, the safety of ZOL treatment is reliable.

Compared with long-term oral drugs, ZOL only needs to be injected once a year, which not only avoids digestive tract adverse reactions caused by oral bisphosphonates, salmon calcitonin, and other drugs but also makes the medication administration more convenient, thereby greatly improving patients' medication compliance. Moreover, with the significant relief of back pain, patients' medication compliance will be further improved.

## 5. Conclusion

In terms of relieving postoperative pain in patients with OVCF, the preoperative ZOL administration combined with PVP surgery was significantly better than the control treatment. Preoperative intravenous infusion of ZOL can reduce bone loss, increase bone density, reduce the risk of refracture, and improve patient quality of life.

## Figures and Tables

**Table 1:** Baseline characteristics of each group.

Variable	ZOL	Conservative	*P* value
Number	121	121	
Gender (female/male)	72/49	81/40	0.27
Age (years)	62.60 ± 7.20	67.45 ± 4.12	0.65
Weight (kg)	67.73 ± 5.11	69.62 ± 6.70	0.46
BMI (kg/m^2^)	26.15 ± 3.21	26.79 ± 5.49	0.96
Fracture levle			
Thoracic	89	101	
Lumbar	57	51	

**Table 2:** Comparison of bone mineral density of the left femoral neck between the two groups before and after the treatment.

	*N*	Before treatment	6 months after treatment	12 months after treatment
ZOL	121	0.41 ± 0.05	0.45 ± 0.05	0.58 ± 0.05^*∗*^
Conservative	121	0.41 ± 0.05	0.43 ± 0.04	0.44 ± 0.05
*P*		0.76	<0.01	<0.01

^*∗*^ *P* < 0.05 ZOL group vs conservative group 6 month after treatment.

**Table 3:** Comparison of PINP and *β*-CTX levels between the two groups before and after the treatment.

	*N*	Before treatment	After treatment		
			1 months	6 months	12 months
P1NP					
ZOL	121	38.85 ± 2.01	28.77 ± 1.89	14.79 ± 1.01^*∗*^	16.53 ± 5.23^*∗*^^*∗*^
Conservative	121	39.76 ± 2.76	34.12 ± 5.41	32.11 ± 4.71	32.76 ± 2.31

*β*-CTX					
ZOL	121	0.47 ± 0.02	0.37 ± 0.01	0.19 ± 0.01^*∗*^	0.27 ± 0.06^*∗*^^*∗*^
Conservative	121	0.48 ± 0.01	0.45 ± 0.02	0.44 ± 0.04	0.45 ± 0.04

^*∗*^ *P* < 0.05 ZOL group vs conservative group 6 month after treatment; ^*∗*^^*∗*^*P* < 0.05 12 months after treatment.

**Table 4:** Complications of the ZOL group and conservative group.

Variable	ZOL	Conservative
New vertebral body fracture *n* (%)	2 (1.7)	13 (10.7)
Bone cement leakage *n* (%)	8 (6.6)	10 (8.3)
Fever	21	
Flu-like symptoms	17	
Myalgia	9	

## Data Availability

All data are included in the manuscript. The datasets used and/or analyzed in the present study are available from the corresponding author upon reasonable request.

## References

[1] Zhong B.-Y., He S.-C., Zhu H.-D. (2017). Risk prediction of new adjacent vertebral fractures after PVP for patients with vertebral compression fractures: development of a prediction model. *Cardiovascular and Interventional Radiology*.

[2] Han S. L., Wan S. L., Li Q. T. (2015). Is vertebroplasty a risk factor for subsequent vertebral fracture, meta-analysis of published evidence?. *Osteoporosis International*.

[3] Camacho P. M., Petak S. M., Binkley N. (2016). American association of clinical Endocrinologists and American College of Endocrinology clinical practice guidelines for the diagnosis and treatment of postmenopausal osteoporosis - 2016. *Endocrine Practice*.

[4] Yang W., Song J., Liang M., Cui H., Chen H., Yang J. (2019). Functional outcomes and new vertebral fractures in percutaneous vertebroplasty and conservative treatment of acute symptomatic osteoporotic vertebral compression fractures. *World Neurosurgery*.

[5] Lou S., Shi X., Zhang X., Lyu H., Li Z., Wang Y. (2019). Percutaneous vertebroplasty versus non-operative treatment for osteoporotic vertebral compression fractures: a meta-analysis of randomized controlled trials. *Osteoporosis International*.

[6] Wang B., Guo H., Yuan L., Huang D., Zhang H., Hao D. (2016). A prospective randomized controlled study comparing the pain relief in patients with osteoporotic vertebral compression fractures with the use of vertebroplasty or facet blocking. *European Spine Journal*.

[7] Zhang L., Zhai P. (2020). A Comparison of percutaneous vertebroplasty versus conservative treatment in terms of treatment effect for osteoporotic vertebral compression fractures: a meta-analysis. *Surgical Innovation*.

[8] Tan H.-Y., Wang L.-M., Zhao L., Liu Y.-L., Song R.-P. (2015). A prospective study of percutaneous vertebroplasty for chronic painful osteoporotic vertebral compression fracture. *Pain Research and Management*.

[9] Zhang Y., Song J., Hou Y. (2019). Clinical research about the improved PVP method in treatment of acute osteoporotic vertebral compression fractures. *Journal of Orthopaedic Surgery (Hong Kong)*.

[10] Ma Y., Wu X., Xiao X. (2020). Effects of teriparatide versus percutaneous vertebroplasty on pain relief, quality of life and cost-effectiveness in postmenopausal females with acute osteoporotic vertebral compression fracture: a prospective cohort study. *Bone*.

[11] Ge J., Cheng X., Li P., Yang H., Zou J. (2019). The clinical effect of kyphoplasty using the extrapedicular approach in the treatment of thoracic osteoporotic vertebral compression fracture. *World Neurosurgery*.

[12] Wang H., Zhang Z., Liu Y. (2018). Percutaneous kyphoplasty for the treatment of very severe osteoporotic vertebral compression fractures with spinal canal compromise. *Journal of Orthopaedic Surgery and Research*.

[13] Clarençon F., Fahed R., Gabrieli J. (2016). Safety and clinical effectiveness of percutaneous vertebroplasty in the elderly (≥80 years). *European Radiology*.

[14] Feng L., Feng C., Chen J. (2018). The risk factors of vertebral refracture after kyphoplasty in patients with osteoporotic vertebral compression fractures: a study protocol for a prospective cohort study. *BMC Musculoskeletal Disorders*.

[15] Yang J. S., Liu J. J., Chu L. (2019). Causes of residual back pain at early stage after percutaneous vertebroplasty: a retrospective analysis of 1,316 cases. *Pain Physician*.

[16] Yan Y., Xu R., Zou T. (2015). Is thoracolumbar fascia injury the cause of residual back pain after percutaneous vertebroplasty? A prospective cohort study. *Osteoporosis International*.

[17] Himelstein A. L., Foster J. C., Khatcheressian J. L. (2017). Effect of longer-interval vs standard dosing of zoledronic acid on skeletal events in patients with bone metastases. *JAMA*.

[18] Aitken D., Laslett L. L., Cai G. (2018). A protocol for a multicentre, randomised, double-blind, placebo-controlled trial to compare the effect of annual infusions of zoledronic acid to placebo on knee structural change and knee pain over 24 months in knee osteoarthritis patients - ZAP2. *BMC Musculoskeletal Disorders*.

[19] Cai G., Laslett L. L., Aitken D. (2018). Effect of zoledronic acid and denosumab in patients with low back pain and modic change: a proof-of-principle trial. *Journal of Bone and Mineral Research*.

[20] Liu Z., Li C. w., Mao Y. f. (2019). Study on zoledronic acid reducing acute bone loss and fracture rates in elderly postoperative patients with intertrochanteric fractures. *Orthopaedic Surgery*.

[21] Hu Z., Man G. C. W., Kwok A. K. L. (2018). Global sagittal alignment in elderly patients with osteoporosis and its relationship with severity of vertebral fracture and quality of life. *Archives of Osteoporosis*.

[22] Hu K. Z., Chen S. C., Xu L. (2018). Comparison of percutaneous balloon dilation kyphoplasty and percutaneous vertebroplasty in treatment for thoracolumbar vertebral compression fractures. *European Review for Medical and Pharmacological Sciences*.

[23] Xu Y., Wang Q., Hou G., Yao H., Zhao H. (2019). A dual-label time-resolved fluorescence immunoassay for screening of osteoporosis based on simultaneous detection of C-terminal telopeptide (*β*-CTX) and aminoterminal propeptide (P1NP) of type I procollagen. *Scandinavian Journal of Clinical and Laboratory Investigation*.

[24] Zuo C. T., Yin D. C., Fan H. X. (2019). Study on diagnostic value of P1NP and *β*-CTX in bone metastasis of patients with breast cancer and the correlation between them. *European Review for Medical and Pharmacological Sciences*.

[25] Zhang J., Zhang T., Xu X., Cai Q., Zhao D. (2019). Zoledronic acid combined with percutaneous kyphoplasty in the treatment of osteoporotic compression fracture in a single T12 or L1 vertebral body in postmenopausal women. *Osteoporosis International*.

[26] Yokoyama K., Kawanishi M., Yamada M., Tanaka H., Ito Y., Kuroiwa T. (2017). Long-term therapeutic effects of vertebroplasty for painful vertebral compression fracture: a retrospective comparative study. *British Journal of Neurosurgery*.

[27] Takahara K., Kamimura M., Moriya H. (2016). Risk factors of adjacent vertebral collapse after percutaneous vertebroplasty for osteoporotic vertebral fracture in postmenopausal women. *BMC Musculoskeletal Disorders*.

[28] Yang C. C., Chien J. T., Tsai T. Y., Yeh K. T., Lee R. P., Wu W. T. (2018). Earlier vertebroplasty for osteoporotic thoracolumbar compression fracture may minimize the subsequent development of adjacent fractures: a retrospective study. *Pain Physician*.

[29] Lee D. G., Park C. K., Park C. J., Lee D. C., Hwang J. H. (2015). Analysis of risk factors causing new symptomatic vertebral compression fractures after percutaneous vertebroplasty for painful osteoporotic vertebral compression fractures. *Journal of Spinal Disorders and Techniques*.

[30] Li H., Wang Y., Wang R. (2020). Effects of rosuvastatin and zoledronic acid in combination on the recovery of senile osteoporotic vertebral compression fracture following percutaneous vertebroplasty. *The Journal of International Medical Research*.

[31] Liu B., Gan F., Ge Y., Yu H. (2018). Clinical efficacy analysis of percutaneous kyphoplasty combined with zoledronic acid in the treatment and prevention of osteoporotic vertebral compression fractures. *Journal of Investigative Surgery*.

[32] Yang L., Du S. (2015). Efficacy and safety of zoledronic acid and pamidronate disodium in the treatment of malignant skeletal metastasis: a meta-analysis. *Medicine*.

[33] Ding Y., Zeng J.-C., Yin F. (2017). Multicenter study on observation of acute-phase responses after infusion of zoledronic acid 5 mg in Chinese women with postmenopausal osteoporosis. *Orthopaedic Surgery*.

